# LOLAweb: a containerized web server for interactive genomic locus overlap enrichment analysis

**DOI:** 10.1093/nar/gky464

**Published:** 2018-06-06

**Authors:** V P Nagraj, Neal E Magee, Nathan C Sheffield

**Affiliations:** 1 School of Medicine Research Computing, University of Virginia, USA; 2 Center for Public Health Genomics, University of Virginia, USA; 3 Departments of Public Health Sciences, Biomedical Engineering, and Biochemistry and Molecular Genetics, University of Virginia, USA

## Abstract

The past few years have seen an explosion of interest in understanding the role of regulatory DNA. This interest has driven large-scale production of functional genomics data and analytical methods. One popular analysis is to test for enrichment of overlaps between a query set of genomic regions and a database of region sets. In this way, new genomic data can be easily connected to annotations from external data sources. Here, we present an interactive interface for enrichment analysis of genomic locus overlaps using a web server called LOLAweb. LOLAweb accepts a set of genomic ranges from the user and tests it for enrichment against a database of region sets. LOLAweb renders results in an R Shiny application to provide interactive visualization features, enabling users to filter, sort, and explore enrichment results dynamically. LOLAweb is built and deployed in a Linux container, making it scalable to many concurrent users on our servers and also enabling users to download and run LOLAweb locally.

## INTRODUCTION

Growing public data sources present both new opportunities and new challenges: they lead to discovery by providing a context for new findings, but this process requires substantial effort and expertise. Linking newly generated data to existing data is a common first step in bioinformatic analytics, and one particularly successful technique is the functional enrichment analysis of gene sets. In this method, a newly discovered set of genes is compared one-by-one to a large public database of gene sets annotated as functionally similar. Any higher-than-expected enrichment provides evidence of a connection from newly generated data to a pre-defined gene set, which in turn ties its functional annotation information to the newly generated gene set. This technique, epitomized by the popular GSEA tool (1), has been fueled by high-quality gene set annotation projects like MSigDB (1), Gene Ontology (2), KEGG Pathway Database (3), and others. These databases synthesize data from various sources into curated gene sets with some annotated biological similarity. Enrichment analysis of gene sets is straightforward, yet powerful, leading to its use in thousands of studies and spawning hundreds of tools and databases built around gene sets (4). These gene set enrichment methods have been pivotal so far in driving connections to external datasets, but they suffer from a key limitation: data must be gene-centric.

As our understanding of the genome has increased, the gene-centric limitation is becoming increasingly important: genes are no longer viewed as monolithic, but as multifaceted. Furthermore, *genes* make up a small part of the genome, and their expression is controlled by hundreds of thousands of cell-type specific functional elements (5,6), which also house the majority of human SNPs with association to disease traits (7). This expanding perspective from the gene-centric to genomic-region-centric has also been propelled by the technological advances of next generation sequencing, which produces data most naturally analyzed in the context of genomic regions. New technologies that assay function of DNA sequences have dramatically increased our ability to study regulatory DNA. We are now accumulating a comprehensive catalog of regulatory elements, driven by independent research labs and large consortium projects such as ENCODE (ENCyclopedia Of DNA Elements) (7) and the IHEC (International Human Epigenome Consortium) (8). This new type of data logically leads to the desire to compare genomic loci rather than genes, leading to new types of analysis.

One such analysis method is *genomic region set enrichment analysis*. This analysis is similar to enrichment analysis of gene sets, but it relaxes the gene-centric viewpoint; instead of using sets of gene symbols, it uses genomic region sets for both query and database. This kind of analysis is now widespread; in a recent review, Dozmorov (9) identified and compared more than a dozen relevant tools, including ReMap (10,11), ChIPseeker (12), GenomeRunner (13), and others. Earlier, we developed an R package called LOLA (Locus OverLap Analysis) (14), which provided a useful, scriptable tool for R programmers to run interactive or automated enrichment analysis of genomic regions on custom databases. LOLA has now proven useful for a variety of biological applications. Here, we present LOLAweb, an R Shiny app and web server for interactive LOLA runs. In addition to a web interface, LOLAweb provides new interactive visualization features to enable LOLA users to explore enrichment results in novel ways. Furthermore, LOLAweb is powered by a robust infrastructure based on Docker Swarm, which enables scalability and portability: Our servers can handle many simultaneous Shiny sessions, and interested users may also run a containerized LOLAweb server on their own custom hardware and using custom databases. These features make LOLAweb a flexible system that will be a valuable research tool with wide application.

## MATERIALS AND METHODS

### LOLA's approach to region set enrichment analysis

The LOLA approach to region set analysis builds upon concepts we developed and validated biologically in previous work (14,15). The LOLA R package (14) has now been used in dozens of publications. LOLA's fundamental unit is the *region set*—a collection of genomic ranges. Region sets may be defined in various ways, including either experimental or computational methods. Some common examples of region sets include the following: annotations of CpG islands; regions of high sequence conservation across species; open chromatin regions defined by DNaseI hypersensitivity; transcription factor binding sites from motif analysis or chromatin immunoprecipitation (ChIP) experiments, *etc*. LOLA uses this type of region set data for both the query and the database. We have already put together a collection of thousands of such region set data from various public data sources, which serve as a database for testing enrichments with a user-provided query set.

LOLA requires three inputs for an analysis: a user query region set, a universe (background) region set, and a curated database with collections of region sets. With these inputs, LOLA identifies the region sets from the database that are most similar to the query set. LOLA does this by comparing the query region set to each database region set and calculates the number of overlapping regions for each pairwise comparison. Along with a similar calculation for the universe region set, LOLA uses the number of overlaps and non-overlaps to build a contingency table, and then uses a Fisher's exact test to assess the significance of the overlap. After computing these statistics for each comparison, LOLA ranks each database region set and provides a ranked summary of the top database sets. This procedure effectively pulls out the region sets in the database that are most similar to the query region set. Now, LOLAweb makes this analysis available on the web, so that users can quickly and easily run a basic region-set analysis without even opening an R session.

### LOLAweb: a Shiny app and web server for interactive LOLA runs

LOLAweb uses LOLA under the hood for statistical analysis. Analogous to LOLA, LOLAweb requires three inputs: one or more query sets, a universe (background) set, and a database. LOLAweb provides a user interface whereby the user provides selections for each of these 3 components. One of the advantages of LOLAweb is ease of use: the database and options for the universe set are both provided by the web server, so the user needs only to bring a single BED-like file to get started on a basic LOLA analysis.

#### User query set

Users upload one or more BED-like files with regions of interest. Users must specify at least *chromosome, start*, and *end* coordinates in the file. These files can be specified with a drag-and-drop interface into the select box.

#### Universe set

The universe represents the restricted background set of regions that could potentially have been included in the query sets, including all regions of interest as well as those that did not get included (*e.g*. ChIP peaks that were not differential). Typically, LOLAweb will yield the best results if the user carefully selects a universe set that is appropriate to the analysis at hand; however, selection of the universe set can be nontrivial and has important implications. A detailed discussion of how to select an appropriate universe is available in the LOLAweb documentation. To lower the barrier for a simple analysis, LOLAweb provides a few basic options for reasonable starter universe sets. First, the user may select an included universe with a drop-down menu. These include simple genome tiling regions and a set of all active regulatory elements as defined by cross cell-type DNase hypersensitivity experiments. Second, if a user uploads more than one query file, the user may choose to automatically build a *restricted universe*, which is the union of the uploaded query sets. This option is particularly applicable to experiments testing differential signals, such as sets of histone modifications that either increase or decrease with some perturbation. These built-in options provide a way for many analysis to get good first-pass results without requiring the user to define a custom universe. Finally, the user may choose to upload a BED-like file to use as a custom universe specific to the problem at hand.

#### Region database

Third, the user must select a region database from among the provided options. LOLAweb provides built-in databases for human (hg19 and hg38 builds) and mouse (mm9 and mm10 builds). Several database choices are available for different reference genomes, and the contents of each database is described in detail in the LOLAweb documentation. We intend to update these databases as new data becomes available. The primary database, which will be useful for most analysis, is the LOLA Core database provided with LOLA (14). This database includes collections of thousands of region sets from many public sources, such as the ENCODE transcription factor binding data (7), DNase hypersensitivity data (16), the Cistrome (17) and CODEX (18) databases, and more. LOLAweb is designed to easily handle custom databases as well, but because of the impracticality of transferring large database files, users who want to use a custom database must run LOLA or LOLAweb locally.

### Assessing the degree of enrichment

LOLA uses three summary statistics to assess the degree of overlap: (i) the *P*-value and (ii) odds ratio from a Fisher's exact test and (iii) the raw number of overlapping regions. Each of these statistics emphasizes a different aspect of the comparison; for example, the number of overlapping regions emphasizes sheer volume of overlapping regions but does not correct for significance, while the odds ratio emphasizes relative enrichment but can be dominated by sets with small numbers of regions. To come up with an aggregate score that shares strengths of each of these statistics, LOLA ranks each pairwise comparison for each of these three statistics independently, and then calculates a combined rank for each region set by assigning it the worst (max) rank among these three. To rank highly in the combined rank, then, requires that a comparison do reasonably well on all three measures, because the worst score is taken. In our experience, this process prioritizes biologically relevant associations and eliminates spurious relationships (14,19).

### Secondary analysis

In addition to the primary analysis of testing enrichment of overlaps, LOLAweb also provides a few other useful general analyses to explore the genomic distributions of the query regions. These include three complementary analyes: a visualization of the distribution of regions across chromosomes; a summary of the distribution of distance from each region to the nearest transcription start site; and a classification of how the query regions are distributed with respect to genic partitions (e.g. proximal promoter versus intergenic versus exon overlap). These plots provide useful general information about the input query sets outside of the context of the primary enrichment analysis.

### Implementation

LOLAweb uses several concepts to minimize computational requirements. First, the back-end processing uses the venerable GenomicRanges family (20) and data.table R packages, employing optimized compiled code for region set storage and vector calculations. LOLA also implements database caching using the simpleCache package (21), making it orders of magnitude faster to re-load large databases after an initial load. These optimizations make analysis fast and memory efficient, requiring only a few minutes of processing time for an analysis with thousands of database region sets.

**Figure 1.**
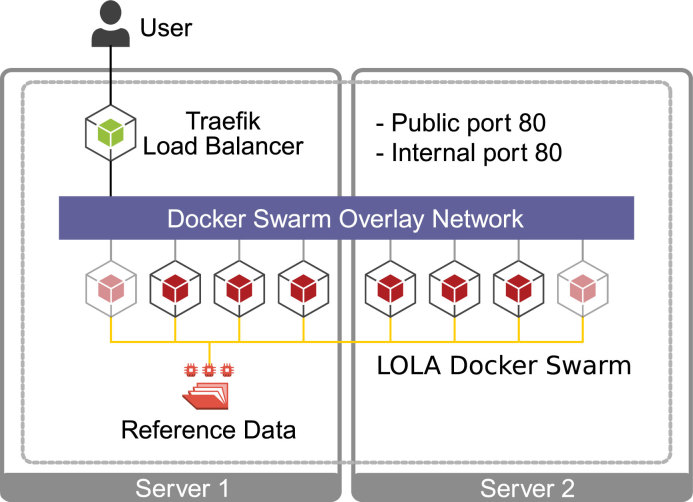
Overview of the LOLAweb architecture. LOLAweb requests are routed from the user to a Traefik load balancer, which allocates Docker containers in a swarm that spans two physical servers. Each Docker container mounts reference data from the host.

LOLAweb uses the R Shiny framework, which serves the application as a single process via the open-source Shiny Server. To support multiple concurrent users, LOLAweb has been containerized using Docker and deployed using Docker Swarm (Figure 1). The swarm spans our hardware, which initially consists of two physical servers, but can be easily scaled up with additional nodes with no necessary changes in software. The Docker stack incorporates a load-balancer using the Træfik container image, which receives user requests, assigns them to idle containers, and associates containers to users through the use of sticky sessions. LOLAweb container images are built hierarchically, with a base layer containing R, Shiny, and other prerequisites, and a LOLAweb image that adds the source code for the app. This approach makes updating LOLAweb fast, as the base layer is typically more stable. When each LOLAweb container launches, it mounts all necessary sample and reference data sets from the host machine to the appropriate locations in the container. This design reduces redundancy and the size of each container. LOLAweb containers process user data against reference data, render the output within minutes, then return to an idle state ready for re-use.

The container approach also simplifies the distribution of LOLAweb. Since Docker images are self-contained applications, LOLAweb can easily be run as a standalone application, or in a Docker Swarm in other environments. The portability of containers and the ability to describe a more complex environment in code means that LOLAweb (i) is reproducible; (ii) can easily handle many simultaneous users; (iii) is scalable as popularity and computing needs grow; and (iv) encourages the sharing of results and methods among researchers. Both the LOLAweb container image and the LOLAweb stack definition are available for download.

## RESULTS

### Interactive result plots and interactive features

**Figure 2.**
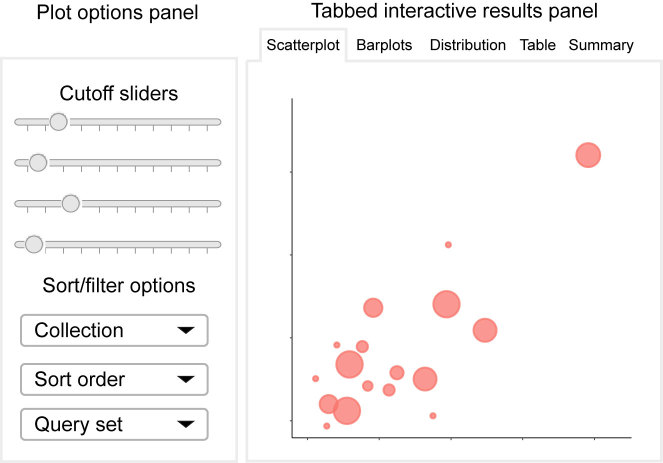
Conceptual diagram of the LOLAweb interactive visualization environment. A display option panel (left side) contains cutoff sliders and select boxes that allow the user to adjust plot display preferences. The interactive results panel (right side) visualizes the top enrichments from the database, among other things and updates on-the-fly based on user input.

After the user has uploaded or selected each of the three required inputs, the server takes just a couple of minutes to run a basic analysis. Once the calculations complete, LOLAweb presents the user with a tabbed interactive R Shiny results viewer (Figure 2). This viewer organizes results into a *Display options* panel and five *Results* tabs:

#### Display options panel

LOLAweb includes interactive sliders that can be adjusted to change the minimum cutoff for enriched entries in the plots. In this way, the user can quickly explore and determine appropriate cutoffs to increase or reduce the number of comparisons contained in the plot, and explore how the different statistics interact with each other. We also provide a ‘master slider’ that operates on the *max rank* metric described earlier. This master slider allows the user to easily modulate the total number of comparisons displayed on the barplots. The user can also adjust the sorting column and filter the plots to display only individual uploaded query sets or only individual collections within the database. Plots are regenerated on-the-fly as the user changes sliders or select boxes, providing an interactive experience that makes it possible to explore the top enrichments.

#### Tab 1: Scatterplot

The primary visual output from LOLAweb is a scatterplot that visualizes data on each of the 3 metrics LOLA uses to assess overlap. Each query-to-database set interaction is a point, with the size of the point corresponding to the number of regions in the overlap. The axes measure the *P-value* and *odds ratio* of each comparison. Thus, large points in the upper-right region of the plot are the best hits for all 3 metrics, and therefore most likely to be biologically relevant. Comparisons that do well in one of the categories show up near the x or y axis, while most of the irrelevant comparisons will accumulate in the lower left part of the plot. The scatterplot uses the plotly package for interactive mouseovers that display detailed information about the comparison when the user identifies an individual point to inspect.

#### Tab 2: Barplots

**Figure 3.**
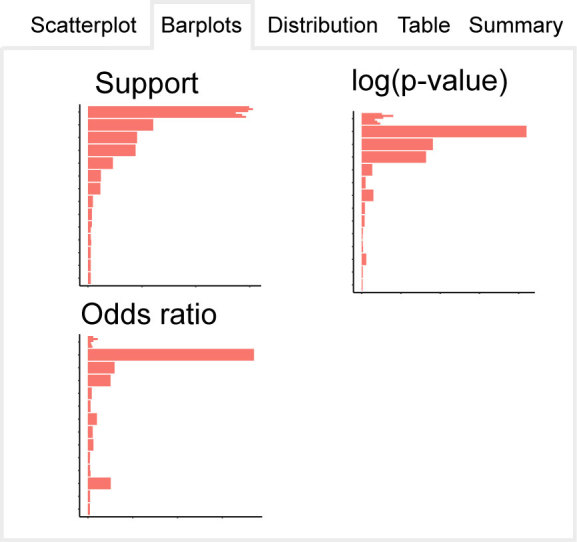
The interactive results panel has several other tabs for exploring the data. This figure shows a conceptual example of the display on the second tab, which depicts barplots that visualize the top enrichments from the database in individual plots for each overlap metric.

To supplement the scatterplot, LOLAweb provides three barplots, one for each of the three statistics assessing overlap (Figure 3). These barplots also dynamically update based on the cutoff sliders, and provide a simpler way to visualize relationships within just one of the statistics. To streamline visualization, we limit the number of comparisons that can show up on each plot.

#### Tab 3: Distribution plots

LOLAweb also provides other summary plots showing how the query regions are distributed across the genome, relative to TSSs, and with respect to genic partitions.

#### Tab 4: Table

In addition to plots, LOLAweb also displays an interactive table showing the top results from the analysis. This results table contains all of the results returned by runLOLA, and is also responsive to the cutoff sliders and sorting selections defined by the user, making it easy to explore the exact comparisons in detail. Furthermore, for collections that are linkable, such as the cross-cell-type DNAse hypersensitivity database (16), the table also provides a hyperlink to detailed web pages that show the characteristics of the particular region set. The table can also be downloaded as a CSV for storage, publication or further analysis within a local R environment.

#### Tab 5: Run summary

The final tab provides metadata regarding the analysis, including the date and elapsed time, the name of the uploaded files, and the version of LOLAweb used to complete the analysis.

Any of the plots may be downloaded to vector-based PDFs for storage, sharing or publication.

### Storage and sharing of results

The results of LOLAweb sessions are cached and given a unique key, allowing users to revisit or share the output URL without having to re-run the original computation. Because the results are relatively small (on the order of megabytes), we can anticipate being able to retain results for a year or more without extensive storage costs, which makes it reasonable for users to share and revisit their results. LOLAweb does not store original data uploaded by the user.

### Comparison to existing tools

The general concept of genomic region set enrichment analysis relaxes the gene-centric constraint of traditional gene set enrichment tools. Popular gene set enrichment tools like GSEA require gene-centric data, which are not directly comparable to the region-centric approach presented here (1). A hybrid approach is taken by another tool called GREAT (22), which maps genomic regions to genes before testing for gene set enrichment. More recent tools have extended this to region sets on both the query and database side, and these tools have been recently reviewed (9). Web servers most similar to LOLAweb include ColoWeb (23) and GenomeRunner Web (24), which both provide interactive, web-based exploration of enrichment analysis results on a region set scale. GenomeRunner also uses the Shiny framework for visualizing results. However, LOLAweb and GenomeRunner fundamentally differ in scope: GenomeRunner is geared toward analysis of SNP datasets, while LOLAweb is targeted toward region-based inputs. GenomeRunner also includes additional analytic methods (i.e. differential regulatory analysis and cell type enrichment), whereas LOLAweb aims to provide a single interface for preparing and summarizing region set overlap, focusing on interactive filtering and visualizations specific to enrichment analysis. ColoWeb differs from LOLAweb in that users select a combination of cell lines and features to compare to the regions of interest (ROI), rather than specifying a preloaded database. ColoWeb measures the strength of association via the density of features in the ROI. LOLAweb quantifies similarity based on overlap with regions in the database, and uses the metrics for overlap (odds ratio, log *P* value, support) to facilitate data exploration. LOLAweb therefore allows a user to visualize multiple features in a single plot and also uses R Shiny to provide interactive filtering based on different measures of statistical overlap. In addition, LOLAweb is unique in providing a container such that users can run local instances, which has been of growing interest in bioinformatics (25). LOLAweb thus complements existing tools with a generic implementation of interactive enrichment analysis in a scalable and portable interface.

## DISCUSSION

The past few years have seen an explosion of interest in understanding the role of regulatory DNA. Studies of regulatory DNA will continue to yield important new insights, and this has led to a rapid increase in production of functional genomic data. These growing data resources have contributed to new approaches to address the challenges with analyzing region-based data, including genomic region set enrichment analysis. To make this analysis more accessible, we have implemented LOLAweb, which provides an intuitive and powerful web interface to allow researchers to connect existing annotation data to newly generated region-set data. Furthermore, LOLAweb serves as a blueprint for developing portable, modular bioinformatics web tools that can be easily run locally on in-house data. We anticipate that LOLAweb will be a useful community resource for interactive analysis of genomic region-set enrichments.

## DATA AVAILABILITY

Web server: http://lolaweb.databio.org. Source code: http://github.com/databio/LOLAweb.
